# Association of physician burnout with perceived EHR work stress and potentially actionable factors

**DOI:** 10.1093/jamia/ocad136

**Published:** 2023-07-20

**Authors:** Ming Tai-Seale, Sally Baxter, Marlene Millen, Michael Cheung, Sidney Zisook, Julie Çelebi, Gregory Polston, Bryan Sun, Erin Gross, Teresa Helsten, Rebecca Rosen, Brian Clay, Christine Sinsky, Douglas M Ziedonis, Christopher A Longhurst, Thomas J Savides

**Affiliations:** Family Medicine, UC San Diego School of Medicine, La Jolla, California, USA; Outcomes Analysis and Scholarship, Information Services, UC San Diego Health, La Jolla, California, USA; Research and Learning, Population Health Services Organization, UC San Diego Health, La Jolla, California, USA; Medicine, UC San Diego School of Medicine, La Jolla, California, USA; UC San Diego Health, La Jolla, California, USA; Medicine, UC San Diego School of Medicine, La Jolla, California, USA; UC San Diego Health, La Jolla, California, USA; Ophthalmology, UC San Diego School of Medicine, La Jolla, California, USA; Medicine, UC San Diego School of Medicine, La Jolla, California, USA; UC San Diego Health, La Jolla, California, USA; Family Medicine, UC San Diego School of Medicine, La Jolla, California, USA; UC San Diego Health, La Jolla, California, USA; Psychiatry, UC San Diego School of Medicine, La Jolla, California, USA; Family Medicine, UC San Diego School of Medicine, La Jolla, California, USA; UC San Diego Health, La Jolla, California, USA; UC San Diego Health, La Jolla, California, USA; Anesthesiology, UC San Diego School of Medicine, La Jolla, California, USA; UC San Diego Health, La Jolla, California, USA; Dermatology, UC San Diego School of Medicine, La Jolla, California, USA; UC San Diego Health, La Jolla, California, USA; Obstetrics and Gynecology, UC San Diego School of Medicine, La Jolla, California, USA; Medicine, UC San Diego School of Medicine, La Jolla, California, USA; UC San Diego Health, La Jolla, California, USA; Family Medicine, UC San Diego School of Medicine, La Jolla, California, USA; UC San Diego Health, La Jolla, California, USA; Medicine, UC San Diego School of Medicine, La Jolla, California, USA; UC San Diego Health, La Jolla, California, USA; Professional Satisfaction, American Medical Association, Chicago, Illinois, USA; Psychiatry, University of New Mexico, School of Medicine, Albuquerque, New Mexico, USA; University of New Mexico Health Sciences and Health System, Albuquerque, New Mexico, USA; Medicine, UC San Diego School of Medicine, La Jolla, California, USA; UC San Diego Health, La Jolla, California, USA; Medicine, UC San Diego School of Medicine, La Jolla, California, USA; UC San Diego Health, La Jolla, California, USA

**Keywords:** professional burnout, physicians, electronic health records, medical informatics, prescription drugs

## Abstract

**Objective:**

Physicians of all specialties experienced unprecedented stressors during the COVID-19 pandemic, exacerbating preexisting burnout. We examine burnout’s association with perceived and actionable electronic health record (EHR) workload factors and personal, professional, and organizational characteristics with the goal of identifying levers that can be targeted to address burnout.

**Materials and Methods:**

Survey of physicians of all specialties in an academic health center, using a standard measure of burnout, self-reported EHR work stress, and EHR-based work assessed by the number of messages regarding prescription reauthorization and use of a staff pool to triage messages. Descriptive and multivariable regression analyses examined the relationship among burnout, perceived EHR work stress, and actionable EHR work factors.

**Results:**

Of 1038 eligible physicians, 627 responded (60% response rate), 49.8% reported burnout symptoms. Logistic regression analysis suggests that higher odds of burnout are associated with physicians feeling higher level of EHR stress (odds ratio [OR], 1.15; 95% confidence interval [CI], 1.07–1.25), having more prescription reauthorization messages (OR, 1.23; 95% CI, 1.04–1.47), not feeling valued (OR, 3.38; 95% CI, 1.69–7.22) or aligned in values with clinic leaders (OR, 2.81; 95% CI, 1.87–4.27), in medical practice for ≤15 years (OR, 2.57; 95% CI, 1.63–4.12), and sleeping for <6 h/night (OR, 1.73; 95% CI, 1.12–2.67).

**Discussion:**

Perceived EHR stress and prescription reauthorization messages are significantly associated with burnout, as are non-EHR factors such as not feeling valued or aligned in values with clinic leaders. Younger physicians need more support.

**Conclusion:**

A multipronged approach targeting actionable levers and supporting young physicians is needed to implement sustainable improvements in physician well-being.

## INTRODUCTION

The unprecedented strain on health care professionals during the COVID-19 pandemic added to substantial preexisting work-related distress among physicians.^1^^,^^2^ Concerns of a parallel pandemic of physician burnout^3^ persist as health systems brace for future surges anticipated from variants, aging population, and staffing shortages. Repeated calls for a national strategy to protect clinicians’ well-being and continued efforts to mitigate burnout have been made.^3^^,^^4^

Work in the electronic health record (EHR) has been identified as an important factor of health care provider clinical time^5^^,^^6^ and physician stress with work and burnout.^7–10^ Physicians in some specialties spend more than half their work day using the EHR to perform multiple clinical and clerical activities, including progress note composition, in-basket message triage and response, order entry, and clinical review.^5^^,^^11^^–^^13^ Time motion studies and analyses of EHR user log have indicated an increase in time allocated for desktop medicine with physicians spending nearly 2 h in the EHR and on other desk work for every hour of direct patient care.^5^^,^^12^^,^^14^^–^^16^ Furthermore, recent data show that the rate of patient emails to providers has increased by more than 50% in the last 3 years.^17^

We aimed to examine the relationship between burnout and stress related to EHR work during the pandemic, measured by physicians’ perception of EHR work stress and potentially malleable measures of their workload in the EHR. The latter included the volume of prescription authorization messages and physicians' choice of being first contact for patient electronic messages versus delegating these messages to a staff pool. We also assessed associations of burnout with professional (specialty), personal (self-care practices)^18^ and organizational factors including perceptions of value alignment, feeling valued, teamwork, and calmness in work environment.^8^

## METHODS

### Participants

All attending physicians in the health system were invited to participate in a confidential survey of their wellness during the height of the COVID-19 pandemic. An invitation to participate in the survey was placed in the physician newsletter on April 17, 2020. The purpose of the survey was described as to seek to understand physician well-being and EHR use during the COVID-19 crisis. Email reminders were sent to various leaders to request their support in cascading the survey invitation to their faculty. Three email reminders were sent to those who did not respond to the survey in May, July, and August 2020. Each participant received a $15 gift certificate. All participants provided informed consent. The survey closed on September 14, 2020. The survey instrument is available under Supplementary Material. The Institutional Review Board approved the study.

Of 1038 eligible attending physicians at the UC San Diego Health System, 627 (60.4%) completed the survey after the online survey verified their eligibility as an attending physician. Comparing demographic characteristics of respondents with administrative data on faculty of the medical school, respondents were more often female (48.3% vs 41.6%). There was a lower percentage of non-Hispanic white physicians among respondents (56.1%) than among School of Medicine faculty (67.1%) and the same percentage of Asians among respondents (22.8%) and among medical school faculty (22.8%). Early career physicians (practicing for ≤15 years) were more represented among respondents (69.7%) than among the faculty (52.6%).

### Variable definitions

Burnout was measured by a validated 5-point scale, with a score of 3 or higher indicating having burnout symptoms.^19^ This scale asks respondents to classify their level of burnout using their own definition of burnout and is widely used in physician burnout research.^8^^,^^20^^–^^22^

Three questions asked about experience with work related to EHR. They were “(1) the amount of time I spend on documentation is, (2) the amount of time I spend on the EHR at home is, (3) the amount of frustration I experience with the EHR during my day is,” each on a scale of 1 (excessive), 2 (moderately high), 3 (satisfactory), 4 (modest), and 5 (minimal/none). These were reverse coded before analysis so that (1) became minimal/none and (5) was excessive. Factor analyses facilitated the creation of a scale to measure EHR stress (described below under “Analytical Approaches”).

We investigated 3 sources of objective EHR work data. The first was vendor generated Signal data (Epic Systems Corporation ©) which were only available on 407 ambulatory care physicians, who represented 65% of the survey participants. Because the study aimed to gain a more comprehensive understanding of EHR workload experienced by multiple specialties, we chose not to use that data source. The second was EHR inbox which had data on 586 (94%) survey respondents. The third was EHR event log in Hyperspace which covered 603 (96%) survey respondents. Twenty-four individuals had no event log data. They were noted as “inactive” in UCSD’s provider information record. The event log data were used in this analysis.

Prior research reported that the volume of inbox messages was not significantly associated with burnout in this sample of physicians.^23^ Therefore, we used alternative objective EHR-related workload measures of the number of prescription authorization messages per day (Rx Messages/day) and whether the respondent had chosen to receive patient messages directly versus delegate to a pool of support staff to screen and triage (Direct MyChart Message). We selected these variables based on their malleability to staffing changes and realignment of practice resources.^24^^,^^25^ We hypothesized that high number of Rx messages would be associated with higher odds of burnout. We further hypothesized that physicians using the pool instead of receiving MyChart messages directly would be associated with lower odds of burnout.

Self-care practices included reports on sleep,^18^ exercise,^26^ and mindfulness practices.^27^ Other individual characteristics were work setting (inpatient vs outpatient), clinical work hours (clinical full time equivalent ≥50% vs <50%), gender (male, female, nonbinary or prefer not to answer). Race was measured by non-Hispanic White, Asian, Black, Hispanic, other or no answer. Due to the small number of respondents among Black, Hispanic, and other races, and those who preferred not to answer the question, we aggregated them into one group representing Black, Hispanic, other, or no answer. Years of medical practice was measured by 1–5, 6–10, 11–15, 16–20, >20 years, and dichotomized to ≤15 years versus >15 years.

Respondents’ perceptions of their work environment were measured by value alignment with their clinical leaders, the atmosphere of their primary work area,^28^ and teamwork efficiency.^29^ Following prior literature,^8^^,^^28^ the feeling valued scale was dichotomized to completely true versus not completely true, the value alignment scale was dichotomized to agree (included strongly agree and agree) versus otherwise, and teamwork efficiency was dichotomized to optimal versus otherwise.

We grouped specialties according to designations by the Centers for Medicare and Medicaid Services (CMS) in which primary care included family medicine, general internal medicine, pediatrics, and geriatrics. We defined COVID-intense specialties according to the frequency of caring for patients with COVID. The COVID-intense specialties included infectious disease, pulmonary and critical care, anesthesiology, hospital medicine, and emergency medicine. Obstetrics and gynecology, and psychiatry were their own groups. Hospital-based specialties included radiation medicine, pathology, and radiology. Other medical and surgical subspecialties included in the study are listed in Table 1.

**Table 1.** Characteristics of survey participants and proportions with burnout symptoms

	Total	Burnout: *N* (%)	
	*N* (%)	Yes	No
*N* (%)	627 (100)	312 (49.8)	315 (50.2)
Perceived EHR stress: mean (SD)	10.5 (2.82)	11.1 (2.80)	9.88 (2.71)
Median (IQR)	11.0 (3.0)	12.0 (4.0)	10.0 (4.0)
Missing: *N* (%)	5 (0.80)	3 (60.0)	2 (40.0)
Prescription authorization messages per day: mean (SD)	0.72 (1.45)	0.84 (1.63)	0.59 (1.23)
Median (IQR)	0.026 (0.68)	0.031 (0.85)	0.025 (0.55)
Missing: *N* (%)	12 (1.9)	5 (41.7)	7 (58.3)
Direct MyChart message			
No	536 (85.5)	268 (50.0)	268 (50.0)
Yes	79 (12.6)	39 (49.4)	40 (50.6)
Missing: *N* (%)	12 (1.9)	5 (41.7)	7 (58.3)
Felt valued			
Not completely true	548 (87.4)	300 (54.7)	248 (45.3)
Completely true	79 (12.6)	12 (15.2)	67 (84.8)
Value alignment with leaders			
Did not agree	247 (39.4)	172 (69.6)	75 (30.4)
Agreed	378 (60.3)	139 (36.8)	239 (63.2)
Missing: *N* (%)	2 (0.3)	1 (50.0)	1 (50.0)
Workspace calm or reasonably busy			
No	131 (20.9)	97 (74.0)	34 (26.0)
Yes	493 (78.6)	213 (43.2)	280 (56.8)
Missing: *N* (%)	3 (0.48)	2 (66.7)	1 (33.3)
Teamwork efficiency			
Not optimal	556 (88.7)	294 (52.9)	262 (47.1)
Optimal	69 (11.0)	17 (24.6)	52 (75.4)
Missing: *N* (%)	2 (0.32)	1 (50.0)	1 (50.0)
Sleep hours			
≥6 h/night	443 (70.7)	196 (44.2)	247 (55.8)
<6 h/night	181 (28.9)	114 (63.0)	67 (37.0)
Missing: *N* (%)	3 (0.48)	2 (66.7)	1 (33.3)
Exercise			
0–3 days/week	386 (61.6)	208 (53.9)	178 (46.1)
≥4 days/week	237 (37.8)	102 (43.0)	135 (57.0)
Missing: *N* (%)	4 (0.64)	2 (50.0)	2 (50.0)
Mindfulness practice			
<1 day per week	336 (53.6)	166 (49.4)	170 (50.6)
≥1 day per week	286 (45.6)	144 (50.3)	142 (49.7)
Missing: *N* (%)	5 (0.80)	2 (40.0)	3 (60.0)
Work setting			
Inpatient	215 (34.3)	112 (52.1)	103 (47.9)
Outpatient	403 (64.3)	194 (48.1)	209 (51.9)
Missing: *N* (%)	9 (1.44)	6 (66.7)	3 (33.3)
Specialty			
COVID-intense^a^	149 (23.8)	82 (55.0)	67 (45.0)
Medical subspecialty	131 (20.9)	55 (42.0)	76 (58.0)
Primary care	113 (18.0)	62 (54.9)	51 (45.1)
Surgery	76 (12.1)	34 (44.7)	42 (55.3)
Hospital-based	69 (11.0)	30 (43.5)	39 (56.5)
Psychiatry	50 (7.97)	30 (60.0)	20 (40.0)
Obstetrics–gynecology	39 (6.22)	19 (48.7)	20 (51.3)
Clinical FTE			
≥50%	506 (80.7)	254 (50.2)	252 (49.8)
<50	103 (16.4)	48 (46.6)	55 (53.4)
Missing: *N* (%)	18 (2.87)	10 (55.6)	8 (44.4)
Gender			
Male	306 (48.8)	131 (42.8)	175 (57.2)
Female	303 (48.3)	169 (55.8)	134 (44.2)
Nonbinary or no answer	18 (2.87)	12 (66.67)	6 (33.33)
Race/ethnicity			
Non-Hispanic White	352 (56.1)	164 (46.6)	188 (53.4)
Asian	143 (22.8)	63 (44.1)	80 (55.9)
Black, Hispanic, other, or no answer	132 (21.1)	85 (64.4)	47 (35.6)
Years in medical practice			
≤15 years	437 (69.7)	244 (55.8)	193 (44.2)
>15 years	187 (29.8)	65 (34.8)	122 (65.2)
Missing: *N* (%)	3 (0.48)	3 (100)	0 (0)

a COVID-intense specialties include infectious diseases, emergency medicine, hospital medicine, pulmonary and critical care, and anesthesiology.

FTE: full time equivalent.

### Analytical approaches

We performed univariate, bivariate analyses, and multivariable logistic regression analyses (dependent variable was burnout symptoms) where covariates included individual characteristics and their perceptions of their workplace. Exploratory and confirmatory factor analyses and diagnostic tests^30–32^ were used to determine the formation of a scale for EHR stress using a sum of responses to the 3 questions on experience with EHR described above. These items loaded with values 0.92, 0.77, and 0.65 onto a distinct factor, respectively. Cronbach’s alpha for the 3 items was 0.79. Parametric item response theory (IRT)^33^ models of the item responses showed a correlation of 0.97 between the sum of the 3 items and the theoretical latent trait scores. Therefore, it was justified for summing the items to measure EHR stress as a numerical score on a scale from 3 (minimal/no stress for all EHR-related items) to 15 (excessive stress for all EHR-related items). We used this summed score to represent global EHR-related stress. All analyses were conducted using R Statistical Software version 4.0.4.

## RESULTS

*EHR-related work*: The average perceived EHR stress score was 10.5 (SD 2.82). The median was 11.0 and the interquartile range (IQR) was 3.0. The average daily prescription authorization messages were 0.72 (SD 1.45) and the median was 0.026 (IQR, 0.68). A small proportion (12.6%) of respondents chose to directly receive MyChart messages rather than delegating those messages to be first read by a staff pool (Table 1).

*Value alignment and feeling valued*: While 60.5% agreed that their values were aligned with their clinical leaders, only 12.6% chose completely true that they felt valued by the organization.

*Participation across specialties*: Of 627 respondents, there were 149 (23.8%) COVID-intense attendings, 131 (20.9%) medical subspecialists, 113 (18.0%) primary care physicians, 76 (12.1%) surgeons, 69 (11.0%) hospital-based, 50 (8.0%) psychiatrists, and 39 (6.2%) OBGYNs. Three hundred three (48.3%) were female (Table 1).

*Variation in burnout across specialties*: After aggregation into CMS’s specialty groups, psychiatry had the highest unadjusted proportion of physicians reporting burnout (60.0%), followed by COVID-intense specialties (55.0%) and primary care (54.9%), obstetrics–gynecology (48.7%), surgeons (44.7%), hospital-based (43.5%), and medical subspecialties (42.0%).

Table 2 presents adjusted odds ratios (ORs) and 95% confidence intervals (CIs). The results suggest that, conditional on covariates in the model, feeling higher level of EHR stress (OR, 1.15; 95% CI, 1.07–1.25), and having more Rx messages (OR, 1.23; 95% CI, 1.04–1.47) were associated with significantly greater odds of burnout. Choosing to directly receive MyChart messages from patients was not significantly associated with burnout (OR, 1.36; 95% CI, 0.75–2.47).

**Table 2.** Factors associated with burnout symptoms

	Adjusted odds ratio	95% CI
EHR stress	1.15***	1.07–1.25
Rx messages/day	1.23*	1.04–1.47
Direct MyChart Message: No (reference)		
Yes	1.36	0.75–2.47
Values alignment with leaders: Agree (reference)		
Disagree	2.81***	1.87–4.27
Feel valued by organization: Completely true (reference)		
Not completely true	3.38***	1.69–7.22
Specialty: Medical subspecialty (reference)		
COVID-intense	2.34**	1.23–4.47
Primary care	2.20*	1.11–4.38
Hospital-based	2.30*	1.04–5.10
Psychiatry	2.61*	1.10–6.35
Surgery	1.90	0.94–3.89
Obstetrics–gynecology	2.76*	1.12–6.90
Gender: Male (reference)		
Female	1.42	0.95–2.12
Race: Non-Hispanic White (reference)		
Asian	0.81	0.51–1.30
Black, Hispanic, or no answer	1.42	0.85–2.38
Years in practice: >15 years (reference)		
≤15 years	2.57***	1.63–4.12
Work setting: outpatient (reference)		
Inpatient	1.28	0.81–2.02
Clinical FTE: ≥50% (reference)		
<50%	1.41	0.82–2.43
Calm or reasonably busy work atmosphere (reference)		
Chaotic work atmosphere	2.05**	1.24–3.43
Team efficiency optimal (reference)		
Not optimal	1.72	0.87–3.50
Slept: ≥6 h/night (reference)		
<6 h/night	1.73*	1.12–2.67
Exercised: ≥4 days/week (reference)		
<4 days/week	1.24	0.83–1.86
Practiced mindfulness for ≥1 day/week (reference)		
<1 day/week	1.10	0.74–1.64
No. of observations	586	

FTE: full time equivalent.

* *P* < .05,

** *P* < .01,

*** *P* < .001.

Physicians’ perceptions of their work environment were significantly associated with burnout. Choosing not completely true to “feeling valued by the organization” (OR, 3.38; 95% CI, 1.69–7.22), disagreed with “values aligned with clinic leaders” (OR, 2.81; 95% CI, 1.87–4.27), “having chaotic workplace atmosphere” (OR, 2.05; 95% CI, 1.24–3.43), having been in practice for ≤15 years (OR, 2.57; 95% CI, 1.63–4.12), and slept for <6 h/night (OR, 1.73; 95% CI, 1.12–2.67) were associated with higher odds of burnout.

Compared to medical subspecialists, COVID-intense specialists had statistically significantly higher odds of burnout (OR, 2.34; 95% CI, 1.23–4.47), as did primary care physicians (OR, 2.20; 95% CI, 1.11–4.38), hospital-based specialists (OR, 2.30; 95% CI, 1.04–5.10), psychiatrists (OR, 2.61; 95% CI, 1.10–6.35), and OBGYNs (OR, 2.76; 95% CI, 1.12–6.90), except surgery subspecialists (OR, 1.90; 95% CI, 0.94–3.89).

## DISCUSSION

The pandemic placed tremendous additional stress on many interconnected parts of the healthcare system and on myriad aspects of physicians’ personal and professional lives. Physician burnout was already a national crisis even before the pandemic. COVID-19 led to a swift pivot to tele-health video and phone visits^34^^,^^35^ which required physicians to learn new ways of communicating with patients, new coding, and new note writing which may have contributed to higher workload in the EHR.^17^^,^^36^ Unsurprisingly, the subjective measure of perceived EHR work stress was significantly associated with burnout. Interestingly, physicians directly receiving MyChart messages, compared to those using a pool to screen messages first, were not associated with having higher odds of burnout. We must caution against prematurely concluding that pools are not helpful, however. Additional research is needed to examine the availability of pools, the decision to use pools, and the qualifications, complexity of the message, and staffing mix (eg, medical assistants, registered nurses, or advance practice providers) of pool members. Anecdotal accounts suggested that some pool members forward most of the messages directly to physicians without taking additional action, therefore diminishing the value of some pools in reducing physicians’ inbox workload.

The finding that prescription authorization message volumes were associated with higher odds of burnout suggests that more support should be provided to physicians. Within their scope of practice, using pharmacists or pharmacy assistants for these tasks has been documented to make a difference.^24^ Our organization has implemented a pharmacist-run refill and prior authorization program aimed at improving physician satisfaction and quality of care in all primary care areas.^37^

The findings of high odds of burnout among physicians who did not feel valued and who perceived their values were not aligned with those of their leaders were consistent with previous literature.^8^ Large national studies suggest that organizations that provide physicians with control over workplace issues and where leaders do “rounds” to engage and align with front-line physicians are more likely to have physicians with higher career satisfaction and lower reported stress or burnout.^21^^,^^38^^,^^39^ Physician–administrator partnership can create practical and sustainable solutions, with attention to appropriate staffing and a focus on needs of all individuals (ie, patients and healthcare professionals) in the practice environment.^40^

On the individual level, early career physicians’ greater odds of burnout could be explained by several possible factors. While we did not specifically ask for details regarding early career physicians, our survey concurs with other pre-COVID-19 studies which identified higher rates of burnout among early or mid-career physicians.^41–43^ The COVID-19 pandemic may have been particularly stressful on this group. These physicians could have young children who were home during that period of the pandemic, requiring caregiving attention as well as support for distance learning. Furthermore, early career physicians may have less control over their schedule and responsibilities due to the hierarchical nature of medical practice and less opportunities for leadership roles which provide increased flexibility of work schedules.^40^^,^^41^ In addition, because older physicians were at higher risk for poor outcomes if infected with COVID-19, some of them did not meet in-person with patients during the pandemic. In support of their older colleagues, early career physicians increased their clinical responsibilities in inpatient or outpatient settings to reduce the risk of exposure to COVID-19 among older colleagues. Therefore, early career physicians might have been more likely to have had direct frontline care of patients (and their families) with COVID-19 and the associated challenges. Lastly, compared to more senior physicians, financial instability may have disproportionately affected some early career physicians who are likely to still be paying off their medical school loans as well as purchasing a first home. Organizational strategies prioritizing early career physicians wellness are needed.^44^ Meaningful support of early career faculty could include mentoring, career coaching, leadership training, and engagement with peers and colleagues who can relate to similar stressors and provide mutual support.^45^

While incremental increase in perceived EHR stress had a statistically significant association with 1.15 times greater odds of burnout, many other statistically significant factors were also associated with burnout. For example, among factors potentially malleable by organizational interventions, not feeling valued by the organization, misalignment of values with leaders, and a chaotic work environment were all associated with high odds of burnout (ORs of 3.38, 2.81, and 2.05, respectively). These findings are consistent with the literature showing that while EHR stress is one significant factor associated with burnout,^8^ it is not the only meaningful factor. Since the CMS and other regulators and payers have modified multiple long-standing policies, including simplifying documentation requirements for billing,^46^ future research should examine the impact of these policy changes on physician wellbeing within the context of other organizational factors.^40^

With respect to actionable findings, we note the significant association of burnout with the number of prescription authorization messages. To the extent possible, prescription authorization can be delegated to staff with appropriate scope of practice to reduce physicians’ workload. Enhancing team-based care and establishing sustainable staffing models consistent with new models of care delivery is urgently needed.^47^ Furthermore, because physicians in all specialties reported burnout, it is important to include both ambulatory care physicians and other specialists in burnout studies and in creation of solutions. To gain better understanding of the relationship between EHR-based work and burnout, we need to use objective EHR work measures that are not limited to ambulatory care physicians. This conclusion is supported by a recent scoping review by Rule et al^48^ which noted increased use of vendor derived EHR work metrics and the continued need for investigator derived measures of EHR work.

Because sleep is recognized as the foundation for health,^49^^,^^50^ it is no surprise that sleeping less than 6 h per night is significantly associated with burnout. While some might view sleep duration as a personal choice, many physicians can attest to the fact that non-face-to-face EHR work in addition to other responsibilities can readily impede that personal freedom. Improving personal sleep practices aside, health care organizations must structure service delivery and work expectations in ways that are conducive to ensuring sufficient sleep among health care professionals, not only for their personal well-being but also for higher quality of patient care.

This study has several limitations. First, the cross-sectional study design cannot establish causation or directionality. Second, the study was done in one academic healthcare system, during the first year of the pandemic, potentially limiting generalizability. Third, the single-item burnout instrument used in this study^19^ correlates closely with the emotional exhaustion subscale of the Maslach Burnout Inventory,^51^ but may not capture the full panoply of burnout symptoms such as depersonalization.^19^^,^^52^ Fourth, other factors that may be related to burnout, such as financial losses, staffing shortage, resilience,^53^ were not directly measured in the survey. Fifth, participation bias, given the nature of subjective self-reporting in the survey, may further limit generalizability. Lastly, constraints in coverage of physicians in multiple specialties in vendor generated EHR work data and EHR audit log data limited this study’s ability to use them in the analyses. Future investigations should continue to identify objective EHR workload measures to inform research and practice improvement.

## CONCLUSION

The most novel finding from this study is that while perceived EHR stress and prescription reauthorization messages are significantly associated with burnout, non-EHR factors (such as not feeling valued or aligned in values with clinic leaders) are also important factors. We need a systems approach that concentrates on the conditions under which individual physicians work and that targets actionable levers such as staff support for prescription reauthorization.^54^ Healthcare organizations continue to face challenges with financial constraints, staffing shortages, and increase in patients due to aging population over the coming years. Indeed, a national survey of physicians showed 62.8% of physicians experienced burnout at the end of the second year of the pandemic.^55^ Organizational leadership can achieve this aim by effectively prioritizing the emotional and mental well-being of its workforce, cultivating a culture of wellness that goes beyond individual personal resilience,^40^ improve engagement and alignment, pay particular attention to younger physicians, implementing policies that value physicians in meaningful ways,^56^ gathering data to drive and evaluate wellness improvements, and lending support for self-care practices.^54^^,^^57^

A meaningful methodological implication of this study is that while vendor generated data on EHR work is a valuable resource for studying ambulatory care physicians,^48^ investigations of perceived EHR stress and actionable EHR work efforts of physicians in diverse specialties need to use data sources that reflect more than ambulatory care physicians. A multipronged approach, supporting the autonomy of physicians and their unique professional and individual needs, with rigorous objective data to monitor progress, is crucial to ensure meaningful and sustainable improvements in physician well-being.

## Supplementary Material

ocad136_Supplementary_Data

## Data Availability

The data underlying this article cannot be shared publicly to protect the privacy of individuals that participated in the study.
