# Supplementary material for: Association of physician burnout with perceived EHR work stress and potentially actionable factors
Source: J Am Med Inform Assoc. 2023 Jul 20;30(10):1665–72. doi: 10.1093/jamia/ocad136 (PMC10531111; doi:10.1093/jamia/ocad136)
Supplement: ocad136_Supplementary_Data [file ocad136_supplementary_data.pdf]

UC San Diego Health

Intro screen

Thank you for your interest in participating in our study! Please take a moment to confirm the following: are you an attending physician at UC San Diego?

Yes, I am an attending physician at UCSD.

No, I am not an attending physician at UCSD.

Consent Block

Welcome to our study of physician wellness and coping with COVID-19. Please click on the link below to access the informed consent in a new screen. You can close the consent form page to return to the survey.

Informed Consent

By clicking “You agree” below you are indicating that you are at least 18 years old, have read this consent form, and agree to participate in this research study.

You agree

You do not agree

Full survey question block

Thank you for agreeing to participate. Please enter your UCSD email address below.

username@health.ucsd.edu

Coping with COVID-19 Survey

The following questions assess the possible impact of COVID-19 on healthcare workers.

	Minimal	Modest	High	Very High

	Minimal	Modest	High	Very High
1) The stress I experienced today is	○	○	○	○

	Not at all	Somewhat	Moderately	To a Great Extent
2) I worry about exposing myself and my family to COVID-19	○	○	○	○

3) Due to the impact of COVID-19, I am experiencing the following:

	Not at all	Somewhat	Moderately	To a Great Extent
Anxiety or depression	○	○	○	○
Work overload	○	○	○	○

	Not at all	Somewhat	Moderately	To a Great Extent
Concerns about childcare	○	○	○	○

If you are feeling severe distress, please contact the UC San Diego Health Healer Education Assessment and Referral Program (HEAR) at hear.ucsd.edu or 858-657-6795, local mental resource, or the national suicide prevention helpline at 1-800-273-8255.

4) How would the following improve your ability to sustain through the COVID-19 crisis?

	Not at all	Somewhat	Moderately	To a Great Extent
Staff or colleague support for inbox	○	○	○	○
Healthy food available at all hours	○	○	○	○

	Not at all	Somewhat	Moderately	To a Great Extent
Personal access to mental health care	○	○	○	○

	Not at all	Somewhat	Moderately	To a Great Extent
5) Being part of the COVID-19 response has increased my sense of meaning and purpose.	○	○	○	○

	Not at all	Somewhat	Moderately	To a Great Extent

	Not at all	Somewhat	Moderately	To a Great Extent
6) I feel valued by my organization.	○	○	○	○

7) What else would you like to tell us about your experience during the COVID-19 crisis?

(Note: anonymous and aggregate responses may be shared with UC San Diego Health's leadership team to inform decision-making.)

Physician Wellness Survey

Please think about your work in general, and answer the following questions.

	Agree strongly	Agree	Neither agree nor disagree	Disagree	Strongly disagree
8) Overall, I am satisfied with my current job.	○	○	○	○	○

9) Using your own definition of “burnout," please choose one of the numbers below*

5 = I enjoy my work. I have no symptoms of burnout.

4

3 = I am beginning to burn out and have one or more symptoms of burnout, e.g. emotional exhaustion.

2

1 = I feel completely burned out. I am at the point where I may need to seek help.

*If you select 1 or 2, please consider seeking assistance – call your healthcare provider or UCSD's HEAR program.

	Agree strongly	Agree	Neither agree nor disagree	Disagree	Strongly disagree
10) My professional values are well aligned with those of my clinical leaders.	○	○	○	○	○

	Poor	Marginal	Satisfactory	Good	Optimal
11) The degree to which my care team works efficiently together is	○	○	○	○	○

	Poor	Marginal	Satisfactory	Good	Optimal
12) My control over my workload is	○	○	○	○	○

	Agree strongly	Agree	Neither agree nor disagree	Disagree	Strongly disagree
13) I feel a great deal of stress because of my job.	○	○	○	○	○

	Excessive	Moderately high	Satisfactory	Modest	Minim

14) The amount of time I spend on documentation is	Excessive	Moderately high	Satisfactory	Modest	Minim
	○	○	○	○	○

	Excessive	Moderately high	Satisfactory	Modest	Minimal/no
15) The amount of time I spend on the electronic medical record (EMR) *at home* is	○	○	○	○	○

		Moderately			

	Excessive	high Moderately	Satisfactory	Modest	Minimal/n
	Excessive	high	Satisfactory	Modest	Minimal/n
16) The amount of frustration I experience with the EMR during my day	○	○	○	○	○

17) Which number best describes the atmosphere in your primary work area?

5 = Calm

4

3 = Busy, but reasonable

2

1 = Hectic, chaotic

	Strongly disagree	Disagree	Neither agree nor disagree	Agree	Agree strongly

	Strongly disagree	Disagree	Neither agree nor disagree	Agree	Agree strongly
18) I am likely to reduce my FTE in the next 12 months.	○	○	○	○	○

19) How true is the following statement about conditions in your principal practice site?

	Not at all true	Somewhat true	Moderately true	Very true	Completely true
Physicians are highly valued.	○	○	○	○	○

20) In the past 2 weeks,

How many hours of sleep did you get each night? [ ]

How many days/week did you exercise for 30 cumulative minutes or more?

How many days/week did you practice mindfulness (e.g. meditation, breathing exercises, gratitude practice)?

Demographic Information

Please respond to the following demographic questions.

21) Please specify your gender.

Male

Female

Non-binary/Third gender

Prefer not to answer

22) What is your race?

Black or African American

Asian

Native American

White

Native Hawaiian or other Pacific Islander

Prefer not to answer

23) What is your ethnicity?

Latino/Hispanic

Not Latino/Hispanic

Prefer not to answer

24) Overall, do you consider yourself to work

Full-time

Part-time

25) Please indicate your percentages of clinical and administrative FTE below.

	0	10	20	30	40	50	60	70	80	90	100
Clinical FTE											
Administrative FTE											

26) In what type of setting do you spend the majority of your clinical time?

27) How many years after training have you been in practice?

1-5 years

6-10 years

11-15 years

16-20 years

More than 20 years

28) Please indicate the Department of your primary appointment at UC San Diego School of Medicine.

29) Please select your Division in the Department of Medicine.

Powered by Qualtrics
